# Effectiveness of Different Organic Solvent Additions to Water Samples for Reducing the Adsorption Effects of Organic Pesticides Using Ultra-High-Performance Liquid Chromatography–Tandem Mass Spectrometry

**DOI:** 10.3390/molecules30010200

**Published:** 2025-01-06

**Authors:** Yucan Liu, Xinyi Xu, Ying Wang, Yan Zhang, Jianbo Lu, Chengbin Liu, Jinming Duan, Hongwei Sun

**Affiliations:** 1 School of Civil Engineering, Yantai University, Yantai 264005, China; 18396679064@163.com (X.X.); w1334513141@163.com (Y.W.); zhangyan-992@163.com (Y.Z.); jianbo98@126.com (J.L.); 2 The Institute of Agro–Food Standards and Testing Technology, Shanghai Academy of Agricultural Sciences, Shanghai 201403, China; 3 Centre for Water Management and Reuse, University of South Australia, Mawson Lakes Campus, Adelaide, SA 5095, Australia; jinming.duan@unisa.edu.au; 4 School of Environmental and Materials Engineering, Yantai University, Yantai 264005, China

**Keywords:** adsorption effect, UHPLC–ESI–MS/MS, organic pesticides, direct injection technique, detection signal intensity, organic solvent, addition ratio

## Abstract

This study systematically investigated the effect of organic solvent addition on the detection signal intensity of 15 organic pesticides in water using ultra-high-performance liquid chromatography–electrospray ionization–tandem mass spectrometry (UHPLC–ESI–MS/MS). The analysis of chromatographic peak area ratios in ultrapure water (UPW) versus 30% methanol (MeOH)–UPW showed that the adsorption effects (AEs, mainly from injection vials with weaker polarity) were the main factor influencing the detection intensity of the organic pesticides. The AEs varied with pesticide type and concentration, especially for those with high logK_ow_ values and longer retention times, such as malathion, triadimefon, prometryn, S–metolachlor, diazinon, and profenofos. Significant differences were observed in the ability of five organic solvents (MeOH, dimethyl sulfoxide, isopropanol, acetonitrile, and acetone) to reduce AEs, with MeOH being the most effective. Optimal solvent ratios were determined to minimize AEs in aqueous solutions. Additionally, plastic injection vials caused greater AEs than glass injection vials, but the addition of organic solvents increased the detection intensity of the analytes for vials of both materials. Density functional theory calculations of the binding energies between pesticides (diazinon, malathion, and S–metolachlor) and vial materials further confirmed the effect of AE on the detection intensity of the analytes. This study showed that the addition of MeOH to real water samples effectively reduced or eliminated the effects of AEs, achieving a good linearity of calibration curves (0.05/0.1–5 μg/L, R^2^ = 0.9853–0.9998), high sensitivity (LOD = 5–32 ng/L), precision (RSD = 1.4–14.5%), and accuracy (average recoveries = 80.6–121.8%). These results provide technical and methodological support for mitigating the effects of AEs on pesticide detection in water using UHPLC–ESI–MS/MS.

## 1. Introduction

Organic pesticides play an important role in reducing crop pests and controlling weeds [1]. However, their widespread use poses significant risks and potential hazards to the ecological environment [2]. Pollution from organic pesticides occurs not only during their application, but also through the contamination of soil and natural water bodies in various ways [3], such as surface runoff and rainwater infiltration. These processes can lead to long-term ecological effects [4]. Organic pesticides can accumulate in the food chain over time and have adverse effects on aquatic organisms and human health [5]. Even at low concentrations, the presence of pesticides in drinking water can pose health risks. Therefore, to ensure the safety of drinking water, it is essential to establish reliable detection and analytical methods for monitoring organic pesticides in surface water and groundwater [6]. This will enable the application of appropriate treatment processes to maintain drinking water quality.

Liquid chromatography–tandem mass spectrometry (LC–MS/MS) in the multiple-reaction monitoring (MRM) mode offers high selectivity [7] and is recognized for its simplicity, cost-effectiveness, and time efficiency [8]. It is widely used for the qualitative and quantitative analyses of trace organic contaminants in water [9]. The direct injection technique (DIT) simplifies the analytical process by injecting samples directly into analytical instruments without extensive pre-treatment [10]. For water-quality testing, the DIT provides rapid results, reduces the risks of sample loss and contamination, and allows the real-time monitoring and adjustment of water treatment processes to improve efficiency and ensure compliance with water-quality standards [11]. It also enables the early detection of potential contaminants, allowing for timely preventative action. In LC–MS/MS analysis, the DIT involves minimal preparation, such as filtration with a syringe filter to remove solid particles and prevent damage to the chromatography column or mass spectrometer. However, factors such as sample pre-treatment, mobile phase composition, instrument stability, and injection vial material can affect the signal intensity and therefore affect detection precision and accuracy. Materials that come into contact with analytes during preparation or detection can adsorb target analytes, particularly hydrophobic organics, reducing the linearity, precision [12], and sensitivity. This effect varies with the binding energy between the analyte and the contact material.

The adsorption effect (AE) is a surface process in which molecules are transferred from a liquid bulk to a solid surface, driven by physical forces or chemical bonding. When LC–MS/MS is used as a detection technique, the intensity of the AE depends on the properties of the analyte and co-eluting compounds [13]. During sample preparation and detection, the AE can result from van der Waals forces between hydrophobic organic analytes (high logK_ow_) and hydrophobic surfaces or specific adsorption under certain conditions [14]. For example, in the quantitative analysis of organophosphorus pesticides (OPPs), pesticide molecules are often adsorbed by container surfaces, reducing the effective concentration and affecting the measurement accuracy. The addition of organic solvents can modify solution properties, reduce adsorption, and improve analytical confidence [15]. Similarly, the AE significantly affects the quantitative analysis of oligopeptides at micromolar concentrations [16], but the addition of organic solvents can effectively reduce analyte loss caused by the AE [17]. The addition of organic solvents to water samples improves the wettability of hydrophobic surfaces [18] and increases the solubility of analytes in the liquid phase. This can effectively reduce or eliminate the adverse effects of AEs on the signal intensity of strong non-polar analytes in LC–MS/MS determination [19] and minimize the adverse effects of membrane adsorption on the detection of organic analytes in water samples [20]. Methanol (MeOH) and acetonitrile (ACN) are often used as modifiers to reduce surface adsorption [19]. Our previous research has shown that the addition of MeOH significantly reduces the effect of the water matrix on the detection of early eluting analytes and eliminates the effect of AEs on the detection of late-eluting analytes [21].

The objectives of this study are to identify the factors causing errors in the detection of organic pesticides in aqueous solutions, to investigate the effect of organic solvent types and ratios on the detection signal intensity of the analytes, to assess the effect of injection vial materials and the mitigating effects of organic solvents on the AE, and to validate the effectiveness of organic solvent addition in reducing the adverse effects of the AE on the detection of organic pesticides in real water samples.

## 2. Results and Discussion

### 2.1. Identifying the Causes of Detection Errors for Organic Pesticides in Water

In this study, 15 mixed standard solutions of organic pesticides (0.1, 0.25, 1, 2.5, 5, 10, 25, 50, and 75 μg/L) prepared with ultrapure water (UPW) were analyzed by a DIT using ultra-high-performance liquid chromatography–electrospray ionization–tandem mass spectrometry (UHPLC–ESI–MS/MS) in the MRM mode. Two calibration curves of the 15 pesticides (the solubility in water is 5–3100 mg/L, which is much higher than 75 μg/L) were fitted in Table 1. Calibration curves were established for two concentration ranges of 0.05/0.1–75 μg/L (R^2^ = 0.9389–0.9993) and 0.05/0.1–5 μg/L (R^2^ = 0.8978–0.9989). The limits of detection (LOD, 8–93 ng/L) and the limits of quantification (LOQ, 27–310 ng/L) for the 15 analytes were relatively high, indicating the low sensitivity of this method for the detection of organic pesticides in water (profenofos was not detectable below 1.0 μg/L). Notably, at low concentrations (<2.5 μg/L), using the 0.05/0.1–5 μg/L calibration curve resulted in larger measurement errors (38.75–433.05%).

Following previous studies [21], 15 pesticide standard solutions (with individual pesticide concentrations of 2.5 μg/L) were prepared in UPW and 30% MeOH–UPW to assess sources of analytical error. The peak area ratio (*PAR*) of the pesticide measured in UPW and 30% MeOH–UPW was calculated (see Figure S1 and Table 1). The *PAR* of cyanazine, simazine, pirimicarb, atrazine, isoproturon, and fenobucarb ranged from 80% to 120%, indicating negligible solvent effects. Carbaryl, diuron (DCMU), and propazine (*PAR* 60–80%) were moderately affected, while malathion, triadimefon, prometryn, S–metolachlor, diazinon, and profenofos (*PAR* < 60%) were strongly affected by the solvent. Overall, longer chromatographic retention times (RTs) correlated with greater solvent effects on the detection intensity of the analytes.

Several factors can affect the detection of analytes. For example, when using LC–MS/MS to detect organic pesticides in water, the structural specificity of pesticides influences their ability to compete for charge during processes, such as solution atomization, droplet flight, solvent evaporation, charge density increase, and Coulombic explosion [22]. Pesticides with high surface activity can capture more charge and generate charged parent ions more efficiently, leading to differences in detection signal intensities between different pesticides [17].

**Table 1:** Main information, calibration curves, LOD, LOQ, and *PAR* for the 15 pesticides in water.

Analyte	MW ^a^ (Da) [23]	WS ^b^ (mg/L) [24]	logK_ow_^c^ [25]	0.05/0.1–75 μg/L		0.05/0.1–5 μg/L		LOD (ng/L) [26]	LOQ (ng/L) [26]	*PAR* (%)
				Calibration Curve	*R* ^2^	Calibration Curve	*R* ^2^			
Cyanazine ^★^	240.69	171 (25 °C)	2.22	*y* = 1151*x* + 1377.6	0.9955	*y* = 1825.1*x* − 351.37	0.9714	22	72	82.79
Simazine ^★^	201.66	6.2 (20 °C)	2.18	*y* = 268.57*x* + 560.54	0.9884	*y* = 487.65*x* − 24.567	0.9912	21	70	119.10
Carbaryl ^★^	201.22	120 (20 °C)	2.36	*y* = 102.79*x* + 23.279	0.9977	*y* = 102.97*x* + 78.273	0.8978	42	139	69.68
Pirimicarb	238.29	3100 (20 °C)	1.70	*y* = 603.56*x* − 51.587	0.9978	*y* = 606.02*x* + 50.821	0.9970	9	29	93.31
Atrazine	215.68	33 (22 °C)	2.61	*y* = 743.72*x* + 967.39	0.9944	*y* = 1112.1*x* − 81.571	0.9956	12	42	96.09
Isoproturon	206.28	65 (22 °C)	2.87	*y* = 1212.9*x* + 2308.8	0.9911	*y* = 1888.2*x* + 20.78	0.9649	5	18	92.82
DCMU ^★^	233.09	37.4 (25 °C)	2.68	*y* = 1666.1*x* + 38.434	0.9993	*y* = 1751.5*x* − 275.39	0.9870	40	134	62.30
Propazine	229.71	5 (20 °C)	2.93	*y* = 863.24*x* + 1062.9	0.9963	*y* = 1408.5*x* − 164.57	0.9922	10	35	77.33
Fenobucarb	207.27	420 (20 °C)	2.78	*y* = 970.58*x* + 688.7	0.9940	*y* = 1304.1*x* − 201.97	0.9830	9	29	81.16
Malathion ^★^	330.36	145 (25 °C)	2.36	*y* = 281.04*x* − 576.01	0.9770	*y* = 147.4*x* + 81.596	0.9741	31	105	15.94
Triadimefon ^★^	293.75	64 (20 °C)	2.77	*y* = 2304*x* + 15548	0.9389	*y* = 9785.5*x* − 279.18	0.9982	50	168	25.79
Prometryn	241.36	33 (22 °C)	3.51	*y* = 1979.3*x* + 4079	0.9889	*y* = 4957.7*x* − 384.07	0.9989	12	40	50.59
S–metolachlor ^★^	283.79	480 (25 °C)	3.13	*y* = 1490.9*x* − 1709.3	0.9979	*y* = 711.81*x* + 40.771	0.9938	15	50	52.91
Diazinon ^★^	304.35	60 (20 °C)	3.81	*y* = 421.5*x* − 774.04	0.9780	*y* = 263.16*x* + 62.339	0.9818	62	207	14.49
Profenofos ^d^	373.63	28 (25 °C)	4.68	–	–	–	–	–	–	16.74

Note: ^a^ Molecular weight; ^b^ water solubility; ^c^ logK_ow_; ^d^ not detected at concentrations below 1.0 μg/L in water; the ranges of calibration curves are 0.1–5 µg/L and 0.1–75 µg/L for pesticides marked with ^★^.

### 2.2. Effect of Organic Solvent Type and Addition Ratio on the Detection of Organic Pesticides

Previous studies have shown that the addition of organic solvents to water samples could effectively reduce the adverse effect of the AE on the detection of the organic pesticides in water [20], and the detection signal intensity of different pesticides may vary with the types and ratios of organic solvents added. Therefore, the effects of five typical organic solvents (MeOH, dimethyl sulfoxide (DMSO), isopropanol (IPA), ACN, and acetone (ACE)) at eight addition ratios (0, 5%, 10%, 20%, 30%, 40%, 50%, and 60%) on the detection of the 15 organic pesticides in water were systematically investigated.

**Figure 1:**
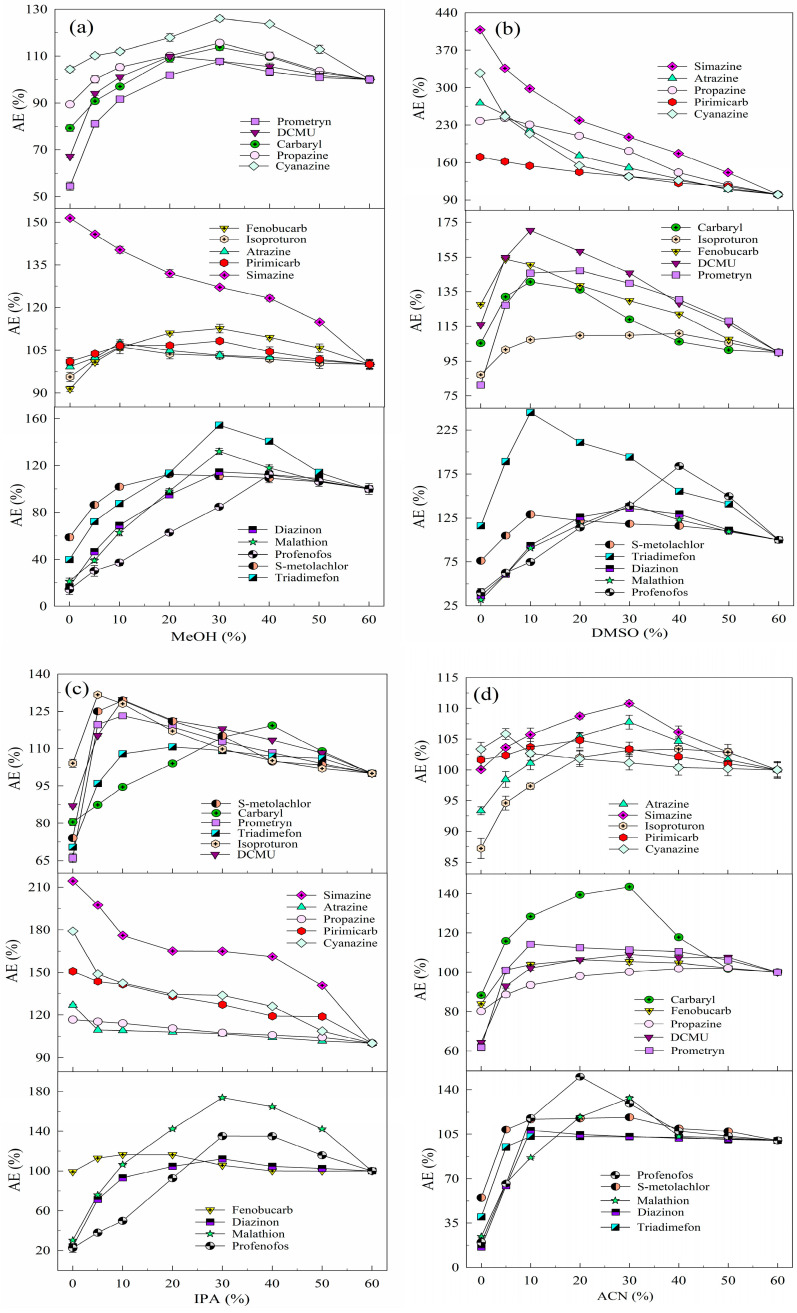

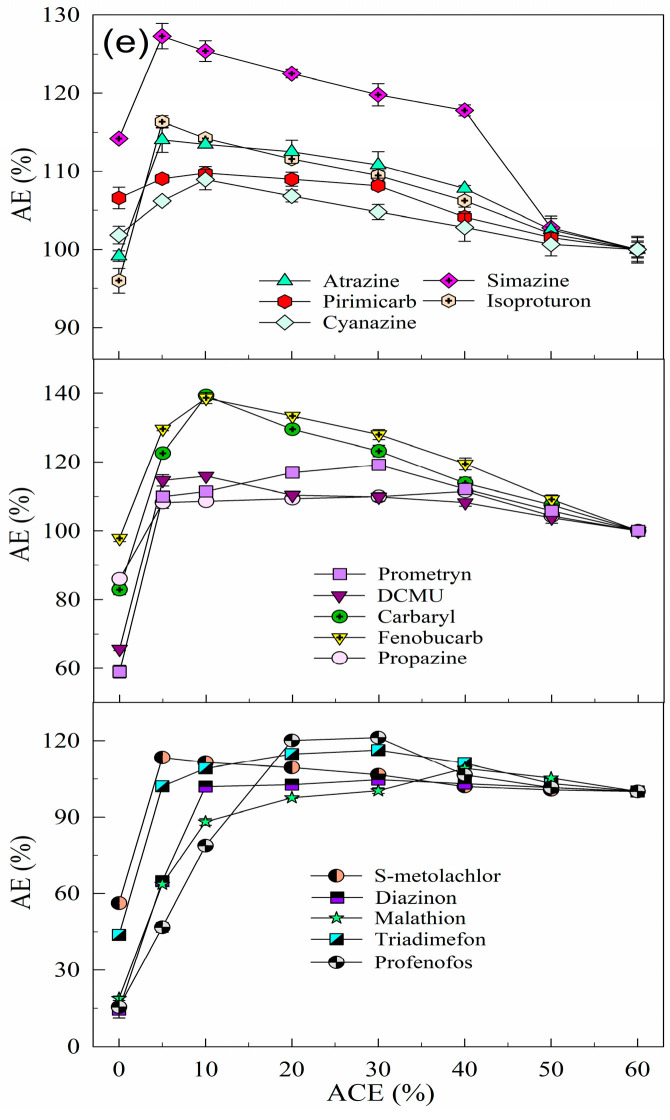
Effect of the organic solvent addition ratio on the AEs of the 15 analytes: (**a**) MeOH, (**b**) DMSO, (**c**) IPA, (**d**) ACN, and (**e**) ACE (2.5 μg/L, the detection signal intensity of the analyte in 60% organic solvent–UPW was 100%).

Previous studies have shown that MeOH improves ESI efficiency for target analytes [27] due to its low surface tension (0.024 N/m) and weak ionization to H^+^ [28], which enhances hydrogenated ion formation and increases chromatographic peak areas. In addition, MeOH increases the non-polarity of the solution, reducing the adverse effect of AE on the detection of the analytes. Analyte standard solutions are usually prepared using the mobile phase composition, and MeOH (mobile phase A) and UPW (mobile phase B) were used as mobile phases in this study. This study systematically investigated the effectiveness of adding MeOH to the aqueous solution in mitigating the effect of AE on detection. The effect of MeOH addition varied between pesticides, and the improvement in the detection signal intensity for the same pesticide depended on the MeOH ratio (see Figure 1a). As the MeOH ratio increased, the *PAR* values of 14 pesticides (excluding simazine) first increased and then decreased, indicating that an optimal MeOH ratio minimizes the adsorption loss of the target analyte. Specifically, 10% MeOH effectively reduced the adverse effect of AE on the detection of isoproturon, atrazine, and pirimicarb; 20% of DCMU and S–metolachlor; 30% of cyanazine, carbaryl, propazine, fenobucarb, malathion, triadimefon, prometryn, and diazinon; and 40% of profenofos. However, higher MeOH ratios reduced the detection signals of the analytes due to solvent effects, where the higher solvent strength of the analyte solution compared to the LC–MS/MS mobile phase caused delayed or forked chromatographic peaks, resulting in abnormal detection signals [29]. In summary, adding 30% MeOH to the aqueous solution significantly increased the detection signal intensity of 14 organic pesticides without significantly affecting their ionization efficiency during ESI after chromatographic elution [30]. Therefore, the use of 30% MeOH–UPW as a solvent for the preparation of mixed standard solutions of organic pesticides can effectively reduce the detection signal problems associated with the AE in UHPLC–ESI–MS/MS.

DMSO, a nonproton dipole solvent soluble in water [31] and organic solvents [32], can dissolve most organic micropollutants at high concentrations and enhance their detection efficiency when added in small amounts. Figure 1b shows the effect of DMSO addition ratios on the detection signal intensity of 15 organic pesticides. The detection signals of cyanazine, simazine, pirimicarb, atrazine, and propazine decreased with increasing DMSO ratios, while other pesticides showed an initial increase in the signal intensity followed by a decrease. Specifically, 10% DMSO effectively reduced the adverse effect of AE on the detection of carbaryl, isoproturon, DCMU, fenobucarb, prometryn, triadimefon, and S–metolachlor; 30% of diazinon and malathion; and 40% of profenofos. This is attributed to DMSO competing with analyte molecules for binding sites on the column head or vial walls, thereby reducing unwanted interactions [10]. However, excessive DMSO reduced the detection signals of all pesticides due to hydrogen bonding between the S=O in DMSO and the O–H in water, altering solution properties [33]. Additionally, high DMSO ratios in real water samples increase the sample volume, thereby decreasing analyte concentrations. In summary, using 10% DMSO–UPW to prepare pesticide mixed standard solutions significantly improved the detection signal intensity of 10 pesticides (carbaryl, isoproturon, DCMU, fenobucarb, malathion, triadimefon, prometryn, S–metolachlor, diazinon, and profenofos) compared to UPW alone. Thus, the addition of 10% DMSO to the aqueous solution effectively reduces AE-related adverse effects on the detection of most organic pesticides.

IPA, known for its solubility in various solvents and higher affinity for lipophilic substances than ethanol [34], has been identified as a stable and simple solvent for high-throughput analytical workflows [35]. In this study, the effect of IPA addition on pesticide detection was similar to that of DMSO. Detection signal intensities of simazine, atrazine, propazine, pirimicarb, and cyanazine decreased with increasing IPA ratios, while those of 10 other pesticides initially increased and then decreased. Specifically, 5% IPA effectively reduced the adverse effect of AE on the detection of isoproturon; 10% of DCMU, fenobucarb, triadimefon, prometryn, and S–metolachlor; 30% of diazinon, profenofos, and malathion; and 40% of carbaryl (see Figure 1c). Overall, the effect of AE on the 10 target pesticides decreased with increasing IPA ratios (0–60%) due to enhanced solvent effects that mitigate AE-related signal suppression [29]. In summary, the use of 10% IPA–UPW as a solvent of pesticide mixed standard solutions effectively enhances detection signal intensities for most pesticides and mitigates AE-related adverse effects on the detection of 15 organic pesticides.

ACN, a moderately polar non-protonic solvent [36] with a high dielectric constant (37.5 F/m), low viscosity (0.34 mPa·s at 25 °C) [37], and surface tension (0.030 N/m), offers advantages when competing with C_18_ column packing and enhancing the separation and solvation of organic pesticide ions [38]. Figure 1d shows that the detection signal intensities of 15 pesticides initially increased and then decreased with increasing ACN ratios in aqueous solutions. Specifically, 5% ACN effectively reduced the adverse effect of AE on the detection of cyanazine; 10% of fenobucarb, triadimefon, prometryn, S–metolachlor, and diazinon; 20% of pirimicarb, DCMU, and profenofos; and 30% of simazine, carbaryl, atrazine, isoproturon, propazine, and malathion. This effect is attributed to ACN disrupting full hydrogen bonds, forming partial hydrogen bonds and altering the microstructure of the aqueous solution as water and ACN molecules cluster by hydrogen bonding. ACN, with its high mass transfer efficiency and strong elution ability, shortens the chromatographic RT of hydrophobic pesticides. However, as the ACN ratio in pesticide solutions increases, the detection signal intensity of target analytes decreases due to the competition of ACN with water molecules, breaking the stable tetrahedral hydrogen bonding network in water [33]. The intensity of the effect of AE on the detection of pesticides depends on their water solubility and solvent properties, which also leads to the discrimination between target analytes. In summary, the use of 20% ACN–UPW to prepare pesticide mixed standard solutions enhances the detection signal intensity of most pesticides, effectively reducing AE-related adverse effects in the analysis of 15 pesticides.

ACE, the simplest saturated ketone [39], is soluble in water and organic solvents, and significantly affects water structure by reducing hydrogen bonding [40]. Figure 1e shows that the detection signal intensity of 15 pesticides initially increased and then decreased with increasing ACE ratios in aqueous solutions. Specifically, 5% ACE effectively reduced the adverse effect of AE on the detection of simazine, atrazine, isoproturon, propazine, and S–metolachlor; 10% of cyanazine, carbaryl, pirimicarb, DCMU, fenobucarb, and diazinon; 20% of profenofos; 30% of prometryn and triadimefon; and 40% of malathion. Excessive ACE reduced the detection signal intensity of target analytes due to competition for solvent binding, inhibition of ion generation, and chromatographic peak areas [22]. In summary, the use of 5% ACE–UPW as a solvent effectively increased the detection signal intensity and reduced the adverse effects of AE for most of the 15 pesticides.

The results show that the addition of the five organic solvents (MeOH, DMSO, IPA, ACN, and ACE) significantly reduced the adverse effects of AE on the detection signal intensity of OPPs, such as malathion, diazinon, and profenofos. This is probably due to reduced interactions between OPP molecules (containing P=O or P=S groups) and active sites in the chromatographic system, increasing the signal intensity compared to UPW. For example, the detection signal intensities of malathion in 30% MeOH–UPW, 30% DMSO–UPW, 30% IPA–UPW, 20% ACN–UPW, and 40% ACE–UPW were 627.50%, 443.00%, 573.27%, 511.20%, 544.48%, and 580.17% of its detection signal intensity in UPW, respectively.

The attenuation of the adverse effects of AE on the detection signal intensity of the target analyte varied with the solvent, with MeOH added to the aqueous solution showing the most significant increase in signal intensity. Pesticides containing functional groups (see Table S1), such as P=O, –O–CO–NH–, –OH, –N=, R–NH–, and –NH–CO–NH–, showed strong matrix enhancement effects, although differences in the polarity and physicochemical properties led to variability, even among compounds with similar groups. OPPs were more susceptible to the AEs, mainly due to their phosphorus content [41]. Variations in AE reduction with DMSO and IPA may be due to differences in solvent and pesticide polarity [42]. In addition, polar pesticides with late chromatographic peak times were also more susceptible to the AEs.

**Figure 2:**
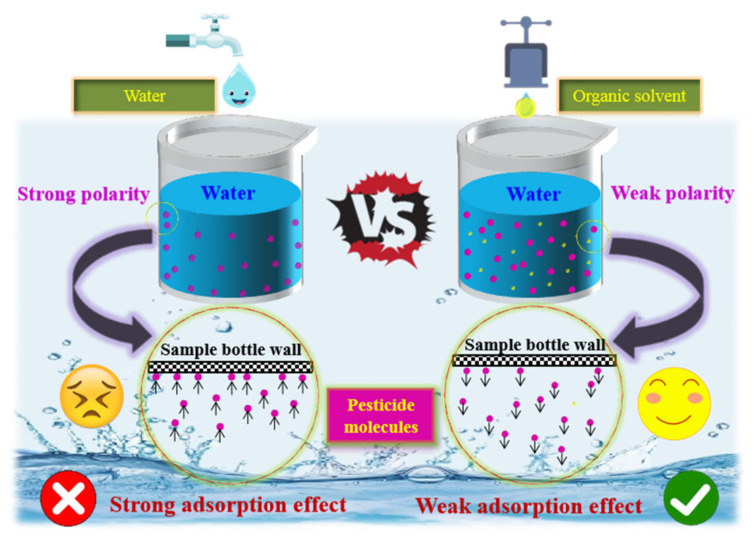
Migration mechanism diagrams of organic pesticides in UPW and organic solvent–UPW.

It is important to note that the polarity of water creates strong intermolecular forces (primarily hydrogen bonds) that stabilize the molecular structure in aqueous solutions. These bonds form stable network structures between water molecules and between water and solute molecules [43], maintaining molecular arrangements and interactions. While hydrogen bonding dominates the water phase, non-polar organic compounds interact with water primarily via van der Waals forces, with no significant polar intermolecular interactions. Consequently, the strong hydrogen bonding in water makes it difficult for non-polar organic molecules to remain in the aqueous phase, causing them to aggregate and migrate to the vial walls due to hydrophobic effects. However, the addition of organic solvents attenuates the adverse effects of AE on the detection signal intensity of organochlorine pesticides (OCPs) by enabling pesticide molecules to migrate from the injection vial walls and disperse into the solvent. This process, shown in Figure 2, does not require the breaking of hydrogen bonds in the organic phase. Instead, van der Waals forces between non-polar molecules stabilize their dispersion, supporting a stable state for non-polar organic molecules in the organic phase.

### 2.3. Effect of Injection Vial Material on the AE of Organic Pesticides and Reduction Strategy

#### 2.3.1. Effect of Injection Vial Material on the AE of Organic Pesticides

**Figure 3:**
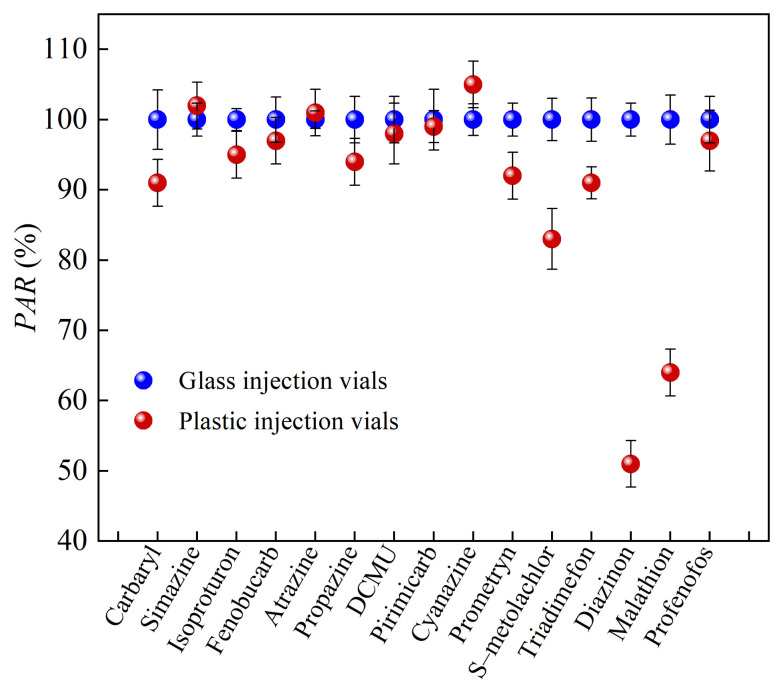
The detection signal intensity of the 15 analytes in glass vials and plastic vials (2.5 μg/L, the detection signal intensity of the analyte in a glass sample vial was 100%).

As detailed in Section 2.1 and Section 2.2, the hydrophobicity of target analytes has a significant effect on the AE, along with the properties of the analytes and the materials of the injection vials. To evaluate the effect of vial material (glass and plastic, with plastic being more hydrophobic than glass) on the detection signal intensity of 15 organic pesticides (2.5 μg/L), UHPLC–ESI–MS/MS was performed in the MRM mode. The *PAR* values obtained using glass vials were set at 100%, and the experimental results are shown in Figure 3.

The detection signal intensities of the most target pesticides were lower in plastic injection vials compared to glass vials, indicating stronger AEs in plastic vials (see Figure 3). Notably, diazinon, malathion, and S–metolachlor experienced the greatest reduction in the signal intensity in plastic vials (*PAR* values were particularly small), highlighting their susceptibility to the AEs in plastic vials compared to glass vials. Diazinon and malathion are both OPPs with strong AEs due to their unique chemical properties. The AE of S–metolachlor is related to its logK_ow_, a parameter that measures the partitioning of a compound into non-polar (octanol) and polar (water) environments. S–metolachlor has a larger logK_ow_ value, indicating that the compound tends to partition into non-polar environments. The plastic injection vial has greater hydrophobicity than the glass injection vial under the same solvent conditions, resulting in the detection signal intensity of strong hydrophobic organics being significantly lower in the plastic injection vial than that in the glass injection vial. Therefore, when using LC–ESI–MS/MS for the detection of organic micropollutants in water samples, it is best to use glass vials to accurately determine the concentration of the target analytes.

**Figure 4:**
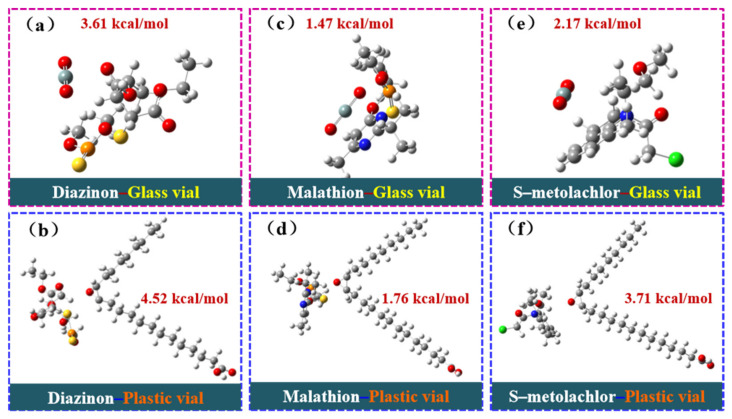
DFT calculation model diagram: (**a**) diazinon–glass vial, (**b**) diazinon–plastic vial, (**c**) malathion–glass vial, (**d**) malathion–plastic vial, (**e**) S–metolachlor–glass vial, and (**f**) S–metolachlor–plastic vial.

The density functional theory (DFT) was used to calculate the binding energies of malathion, S–metolachlor, and diazinon with the injection vial wall in polypropylene plastic vials (composite structure using polypropylene as a computational model) and glass vials (SiO_2_ is the main component and is used as a computational model), respectively. As shown in Figure 4, the binding energy values were obtained by substituting them into the binding energy calculation formula (1). According to the results of the binding energy calculation, the order of binding energy is diazinon–plastic vial (4.52 kcal/mol) > diazinon–glass vial (3.61 kcal/mol), malathion–plastic vial (1.76 kcal/mol) > malathion–glass vial (1.47 kcal/mol), S–metolachlor–plastic vial (3.71 kcal/mol) > S–metolachlor–glass vial (2.17 kcal/mol). It should be noted that the higher the binding energy, the more stable the adsorption and binding between the target analyte and the vial wall. As shown by the calculation results, the binding energy of pesticides in plastic vials is high, and pesticides in plastic vials are more easily adsorbed to the vial wall, whereas pesticides in glass vials are more easily to be distributed uniformly in solution. Therefore, pesticides in plastic vials are more susceptible to the adverse effects of AE and are difficult to detect, whereas pesticides in glass vials are relatively less affected by the AE and are easier to detect.

(1) $$\Delta E=E\left(A-W\right)-E\left(A\right)-E\left(W\right)$$

#### 2.3.2. Effect of Organic Solvent Addition on the AEs for Pesticides in Injection Vials of Different Materials

The 15 pesticides (2.5 μg/L) were determined in glass and plastic injection vials with the addition of 30% organic solvent, and the effects of the organic solvent on the AEs of the target analytes in the glass and plastic injection vials are shown in Table S2 and Table S3, respectively. As shown in Table S2, the addition of 30% MeOH, 30% ACN, and 30% ACE in the solvent to the prepared mixed standard solution of the 15 pesticides for the glass material injection vial effectively reduced the effect of AE on the detection signal intensity of most of the organic pesticides; when 30% DMSO was added, the detection signal intensity of cyanazine, simazine, atrazine, and propazine was greater; when 30% IPA was added, the detection signal intensity of simazine was greater. As shown in Table S3, when 30% of MeOH, 30% ACN, or 30% ACE was added to the plastic injection vials, the adverse effects of AE on the detection signal intensity of most organic pesticides were effectively attenuated; the detection signal intensities of cyanazine, simazine, carbaryl, pirimicarb, atrazine, and propazine were greater with 30% DMSO; and the detection signal intensities of carbaryl, simazine, and cyanazine were greater with 30% IPA.

A comparison of Tables S2 and S3 shows that DMSO significantly reduces the adverse effects of AE on the detection signal intensity of carbamate pesticides and OCPs in plastic injection vials. Furthermore, the addition of organic solvents increased the detection signal intensity of target pesticides in both glass and plastic vials to varying degrees, demonstrating that organic solvents effectively mitigate the effects of AE on the detection signal intensity of organic pesticides.

### 2.4. Method Validation

The linearity, precision, and sensitivity of the proposed method were validated using mixed standard solutions of the 15 pesticides prepared with 30% MeOH–UPW as the solvent. Detailed methods are presented in Text S1. The linearity of the calibration curve for the 15 pesticides was assessed by plotting the triplicate average chromatographic peak areas against their concentrations, with *R*^2^ values provided in Table 2.

After the addition of 30% MeOH to real water samples, 14 pesticides showed excellent linearity (*R*^2^ ≥ 0.9963), while profenofos had a slightly lower *R*^2^ of 0.9853. This method also showed good repeatability, with intra-day relative standard deviations (RSD) ranging from 1.9% to 9.5% and inter-day RSD from 3.2% to 14.5% (see Table 2). In addition, the sensitivity of the proposed method was evaluated using the US EPA method [26], yielding an LOD of 5–32 ng/L and an LOQ of 16–106 ng/L (see Table 2). Standards prepared with 30% MeOH–UPW had significantly lower LOD and LOQ values compared to those prepared with UPW alone (see Table 1), demonstrating higher sensitivity for the detection of organic pesticides. Finally, the concentrations of 15 pesticides in four real water samples (see Table S4) at two addition levels were determined by UHPLC–ESI–MS/MS, and recovery rates were calculated. The average recoveries were in the ranges of 80.6–121.8% at 0.25 μg/L and 84.5–115.6% at 2.5 μg/L, indicating that the proposed method has high accuracy. The addition of 30% MeOH effectively eliminated the AEs and further improved the detection accuracy of the analytes (see Table S5).

## 3. Materials and Methods

### 3.1. Reagents and Materials

Detailed information on the reagents and materials can be found in Text S2.

### 3.2. Preparation of Solutions

A mixed standard stock solution of the 15 pesticides was prepared at a concentration of 250 µg/L for each analyte. The pesticide mixed standard solutions in the range of 0.05 or 0.1–75 µg/L were prepared freshly every day with UPW by diluting the pesticide mixed standard stock solution. The pesticide mixed standard solutions containing *x*% MeOH–UPW were prepared in the automatic injection vial of the UHPLC–ESI–MS/MS by diluting the pesticide mixed standard stock solution with *x*% MeOH (*x*% = 0%, 5%, 10%, 20%, 30%, 40%, 50%, and 60% V_MeOH_/V_total_) and (100–*x*)% UPW. The pesticide mixed standard solutions of *x*% DMSO–UPW, *x*% IPA–UPW, *x*% ACN–UPW, and *x*% ACE–UPW were prepared as above. In Section 2.2, the concentration of each pesticide in *x*% organic solvent–UPW was 2.5 μg/L and was analyzed by UHPLC–ESI–MS/MS in the MRM mode.

### 3.3. Instrumentation and Operating Parameters

The instrumentation and operating parameters are presented in Text S3. The MRM conditions and the quantitative ion extraction chromatograms of the 15 pesticides are shown in Table S6 and Figure S2, respectively.

### 3.4. Experimental Methodology

#### 3.4.1. Calculation of the Peak Area Ratio

(1) To investigate the reasons affecting the detection signal intensities of the target pesticides, the chromatographic peak area of the pesticides in 30% MeOH–UPW at a concentration of 2.5 μg/L for each pesticide was taken as 100%, and the *PAR* of the target pesticides was the ratio of the chromatographic peak area of the pesticide measured in UPW and 30% MeOH–UPW.
  (2) $$PAR\;\left(\%\right)=\frac{\mathrm{Chromatographic}\;\mathrm{peak}\;\mathrm{area}\;\mathrm{of}\;\mathrm{the}\;\mathrm{pesticide}\;\mathrm{in}\;\mathrm{UPW}\;}{\mathrm{Chromatographic}\;\mathrm{peak}\;\mathrm{area}\;\mathrm{of}\;\mathrm{the}\;\mathrm{pesticide}\;\mathrm{in}\;30\%\;\mathrm{MeOH}–\mathrm{UPW}}\times 100$$
(2) To investigate the effect of injection vial material, the detection signal intensity of 15 pesticides was first measured at a concentration of 2.5 μg/L for each pesticide in glass and plastic material injection vials, and the *PAR* of the target pesticide in plastic injection vials was calculated to indicate the effect of injection vial material on the detection signal intensity of the analyte, based on the peak area of the analyte measured in glass injection vials as 100%.
  (3) $$PAR\;\left(\%\right)=\frac{\mathrm{Chromatographic}\;\mathrm{peak}\;\mathrm{area}\;\mathrm{of}\;\mathrm{the}\;\mathrm{pesticide}\;\mathrm{in}\;\mathrm{plastic}\;\mathrm{vials}\;}{\mathrm{Chromatographic}\;\mathrm{peak}\;\mathrm{area}\;\mathrm{of}\;\mathrm{the}\;\mathrm{pesticide}\;\mathrm{in}\;\mathrm{glass}\;\mathrm{vials}}\times 100\%$$

#### 3.4.2. Calculation of the AE

The AE (%) is the ratio of the chromatographic peak area of the target pesticide (2.5 μg/L) measured in *x*% organic solvent–UPW (B) to the chromatographic peak area of the analyte (2.5 μg/L) measured in 60% organic solvent–UPW (A).

(4) $$\mathrm{AE}\;\left(\%\right)=\frac{B\;}{A}\times 100\%$$

## 4. Conclusions

When using UHPLC–ESI–MS/MS for the detection of organic pesticides in water, the injection vial in contact with the target analyte would adsorb the analytes to varying degrees, resulting in a decrease in the detection signal intensity of the analyte. The addition of organic solvents to the water samples can increase the hydrophobicity of aqueous solutions, thereby reducing the adverse effect of AE on the detection signal intensity of the analyte. Therefore, the effects of adding five typical organic solvents in different ratios to a pesticide mixed standard solution on the AE for the detection of 15 organic pesticides by UHPLC–ESI–MS/MS were systematically investigated. The main conclusions of this study are as follows:

(1) The main reason affecting the detection signal intensity and errors at low concentrations for the 15 pesticides was the AE, which varied depending on the pesticide type and concentration. Among them, the AE had a significant effect on the detection for the late-eluting analytes.
(2) Different organic solvents have different effects on reducing the effect of AE on the detection signal intensity of the organic pesticide in aqueous solutions. All five organic solvents can reduce the effect of AE on the detection signal intensity of OPPs to varying degrees, whereas DMSO and IPA specifically reduce the effect of AE on the detection of OCPs.
(3) The effectiveness of reducing the AE varies with different addition ratios of organic solvents. Among them, 30% MeOH, 10% DMSO, 10% IPA, 20% ACN, and 5% ACE can effectively reduce the effect of AE on the detection signal intensity of a variety of organic pesticides in aqueous solutions, with MeOH showing the best performance.
(4) The material of the injection vial has a significant effect on the detection intensity of different analytes in aqueous solutions, and most pesticides in plastic vials show a greater effect of AE compared to glass vials. The addition of organic solvents effectively reduces the adverse effect of AE on the detection signal intensity of the analytes in different vial materials.
(5) The results of the DFT calculations show that the binding energy between analytes and vial materials determines the AE intensity, resulting in lower detection signals for hydrophobic compounds in plastic vials. Therefore, glass vials are recommended for the UHPLC–ESI–MS/MS analysis of hydrophobic organics to minimize the AEs.
(6) The addition of MeOH to real water samples effectively reduces or eliminates the adverse effects of AE on the detection signal intensity of late-eluting analytes. A matrix-matched calibration curve with 30% MeOH was successfully established for the analysis of the 15 pesticides. The proposed method has good linearity (*R*^2^ = 0.9929–0.9996), precision (RSD = 1.4–14.5%), accuracy (average recovery = 80.6–121.8%), and sensitivity (LOD = 5–32 ng/L).

## Figures and Tables

**Table 2:** Linear ranges, linear correlation coefficients (*R*^2^), sensitivity, and precision for the 15 pesticides in the proposed method (30% MeOH–UPW as the solvent).

Analyte	Linear Range (μg/L)	*R* ^2^	LOD (ng/L)	LOQ (ng/L)	RSD (%)			
					Intra–Day (n = 5)		Inter–Day (n = 5)	
					0.25 μg/L	2.5 μg/L	0.25 μg/L	2.5 μg/L
Cyanazine	0.1–5	0.9969	18	60	2.2	3.7	7.5	5.8
Simazine	0.1–5	0.9965	25	81	3.6	3.2	8.9	5.6
Carbaryl	0.1–5	0.9969	29	94	4.2	2.6	7.6	4.0
Pirimicarb	0.05–5	0.9995	8	25	5.9	2.3	8.0	6.2
Atrazine	0.05–5	0.9991	12	40	4.7	3.2	5.8	3.5
Isoproturon	0.05–5	0.9976	5	16	7.9	5.4	5.7	4.3
DCMU	0.1–5	0.9963	25	83	5.8	3.1	8.2	6.8
Propazine	0.05–5	0.9998	8	26	6.2	3.8	6.3	4.2
Fenobucarb	0.05–5	0.9973	7	23	4.4	1.9	5.2	3.4
Malathion	0.05–5	0.9986	5	16	5.7	3.2	5.6	3.8
Triadimefon	0.05–5	0.9990	13	43	6.2	3.4	7.2	6.7
Prometryn	0.05–5	0.9997	6	20	8.6	5.7	8.5	5.5
S–metolachlor	0.05–5	0.9965	8	26	7.7	4.5	7.2	5.8
Diazinon	0.05–5	0.9979	9	29	3.2	2.5	6.4	4.6
Profenofos	0.1–5	0.9853	32	106	9.5	8.8	14.5	10.1

## Data Availability

Data are contained within the article.

## Supplementary Materials

The following supporting information can be downloaded at: https://www.mdpi.com/article/10.3390/molecules30010200/s1, Text S1: Method validation; Text S2: Reagents and materials; Text S3: Instrumentation and operating parameters; Figure S1: The ratio of the chromatographic peak area of the pesticide in UPW and 30% MeOH–UPW; Figure S2: Quantitative ion extraction chromatograms of the 15 pesticides in the MRM mode; Table S1: The name, molecular formula, and structural formula of 15 pesticides; Table S2: The effect of 30% organic solvents on the AEs of the analytes in the glass injection vial; Table S3: The effect of adding 30% organic solvents on the AEs of the 15 analytes in the plastic injection vial; Table S4: Water-quality index of real water samples; Table S5: Recoveries of the 15 pesticides added in the real water samples; Table S6: The name, retention time, and MRM conditions of the 15 pesticides.
