# Supplementary material for: Effectiveness of Different Organic Solvent Additions to Water Samples for Reducing the Adsorption Effects of Organic Pesticides Using Ultra-High-Performance Liquid Chromatography–Tandem Mass Spectrometry
Source: Molecules. 2025 Jan 6;30(1):200. doi: 10.3390/molecules30010200 (PMC11722058; doi:10.3390/molecules30010200)
Supplement: Supplementary file 1 [file molecules-30-00200-s001.zip › molecules-3345636-supplementary.pdf]

Supplementary Materials

Effectiveness of Different Organic Solvent Additions to Water Samples for Reducing the Adsorption Effects of Organic Pesticides Using Ultra-High-Performance Liquid Chromatography–Tandem Mass Spectrometry

Yucan Liu [1,*], Xinyi Xu [1], Ying Wang [1], Yan Zhang [1], Jianbo Lu [1], Chengbin Liu [2,*], Jinming Duan [3] and Hongwei Sun [4,*]

[1] School of Civil Engineering, Yantai University, Yantai 264005, China; 18396679064@163.com (X.X.); w1334513141@163.com (Y.W.); zhangyan-992@163.com (Y.Z.); jianbo98@126.com (J.L.)
[2] The Institute of Agro–Food Standards and Testing Technology, Shanghai Academy of Agricultural Sciences, Shanghai 201403, China
[3] Centre for Water Management and Reuse, University of South Australia, Mawson Lakes Campus, Adelaide, SA 5095, Australia; jinming.duan@unisa.edu.au
[4] School of Environmental and Materials Engineering, Yantai University, Yantai 264005, China
* Correspondence: liuyucan@ytu.edu.cn (Y.L.); liuchengbin@saas.sh.cn (C.L.); sunhw@ytu.edu.cn (H.S.); Tel.: +86-158-5358-8482 (Y.L.); +86-152-0198-2770 (C.L.); +86-156-6690-5528 (H.S.)

Contents

Text S1 Method validation 3

Text S2 Reagents and materials 5

Text S3 Instrumentation and operating parameters 6

Figure S1 The ratio of the chromatographic peak area of the pesticide in UPW and 30% MeOH–UPW (2.5 μg/L). 8

Figure S2 Quantitative ion extraction chromatograms of the 15 pesticides in the MRM mode (2.5 μg/L). 9

Table S1 The name, molecular formula, and structural formula of 15 pesticides. 10

Table S2 The effect of 30% organic solvents on the AEs of the analytes in the glass injection vial. 12

Table S3 The effect of adding 30% organic solvents on the AEs of the 15 analytes in the plastic injection vial. 14

Table S4 Water-quality index of the real water samples. 15

Table S5 Recoveries of the 15 pesticides added in the real water samples. 16

Table S6 The name, retention time, and MRM conditions of the 15 pesticides. 17

Text S1 Method validation

A polyethersulfone (PES) syringe filter (0.22 μm) was first used to filter the real water sample taken from natural water. Then, 30% MeOH and the 15 pesticides were added sequentially to the real water sample. Finally, the effectiveness of the the proposed method was determined by detecting the pretreated sample using UHPLC–ESI–MS/MS.

(1) Linearity

A calibration curve was established for the 15 pesticides using seven concentration ranges from 0.05 or 0.1 to 5 μg/L in UPW. The linearity of the calibration curve was evaluated by plotting the values of the triplicate average of the detected chromatographic peak areas against the concentrations of the 15 pesticides.

(2) Precision

The precision, or the repeatability of the method, was determined by calculating the relative standard deviation (RSD) of the detection signal intensities for the 15 pesticides at two spiked concentrations of 0.25 μg/L and 2.5 μg/L in 30% MeOH–UPW. The intra–day RSD was calculated based on five replicate measurements at each of the two concentrations within 1 day.

(3) Sensitivity

The LOD and LOQ for the 15 pesticides were measured and calculated using the method proposed by the United States Environmental Protection Agency (US EPA) to evaluate the sensitivity of the proposed method.

(4) Accuracy

The accuracy of the proposed method was evaluated using the standard addition method, in which two surface water samples and two groundwater samples were spiked with a mixture of the 15 pesticides at concentrations of 0.25 μg/L and 2.5 μg/L, respectively. The concentrations of the 15 pesticides in the four real water samples at the two concentration levels were then determined by UHPLC–ESI–MS/MS, and the recovery rates of the 15 pesticides were calculated.

Text S2 Reagents and materials

Organic solvents such as methanol (MeOH), dimethyl sulfoxide (DMSO), isopropanol (IPA), acetonitrile (ACN), and acetone (ACE) were purchased from Merck Chemicals (Darmstadt, Germany) with HPLC grade purity. Pesticides such as atrazine (98.8%), fenobucarb (97.4%), isoproturon (99.9%), profenofos (98.0%), prometryn (98.8%), S–metolachlor (98.4%), triadimefon, (99.7%), and malathion (99.7%) were purchased from Sigma–Aldrich Corporation (Bellefonte, PA, USA). The pesticides such as carbaryl (99.5%), simazine (99.0%), diazinon (99.5%), and diuron (DCMU, 99.6%) were purchased from AccuStandard Inc. (New Haven, CT, USA). Propazine (99.0%), pirimicarb (98.5%), and cyanazine (99.9%) were purchased from Dr. Ehrenstorfer GmbH (Augsburg, Germany). Glass injection vials and polyethersulfone (PES) syringe filters (0.22 μm) were purchased from the Waters Corporation (Milford, MA, USA). Plastic injection vials were purchased from Beijing J&K Scientific (Beijing, CHN). 10 mL glass syringes were purchased from Shanghai Feige Abrasive & Sharpener Co., Ltd (Shanghai, CHN).

Text S3 Instrumentation and operating parameters

Chromatographic separation was performed using an ACQUITY^{TM} UPLC system (Waters Corporation, Milford, MA, USA) equipped with an ACQUITY^{TM} UPLC BEH C8 column (2.1 mm × 100 mm, 1.7 μm particle size, Waters). Detection was carried out using a TQD triple quadrupole mass spectrometer with electrospray ionization (ESI) in positive multiple reaction monitoring (MRM) mode (Waters Corporation, Milford, MA, USA). Masslynx 4.1 was used for the real–time instrument control, data acquisition and processing. The UPW (18.2 MΩ·cm, TOC ≤ 1 μg/L) used in the experiments was produced by a Purelab Ultra Analytic system (Elga, Bucks, UK). The mobile phase consisted of MeOH (mobile phase A) and UPW (mobile phase B) at a flow rate of 0.2 mL/min. The elution gradient of the chromatograph was 25% A retained for 1 min, then increased to 100% A within 12 min and retained for 2 min, finally decreased to 25% A and equilibrated for 3 min, with a total sample circulation time of 18 min. The injection volume of the autosampler was 10 μL. The temperatures of the column and the sample manager were set at 35 °C and 25 °C, respectively. The needle of the sample injection syringe was rinsed automatically after each injection to eliminate interference between injections. The needle was rinsed three times with 600 μL of weak wash solvent (ACN/UPW, 10:90, V/V) and 200 μL of strong wash solvent (ACN/UPW, 90:10, V/V) after each rinsing cycle.

By optimizing the instrument parameters, the optimum capillary voltage was set at 3.5 kV and the optimum cone voltage (CV) was determined for each analyte (see Table S4). The detailed determination information of the ‘transition selection’ is as

follows: in order to optimize the MS/MS measurement parameters in the data acquisition systems for the selection of precursor ions of each analyte, 15 pesticides (3 mg/L) were tested individually in the full scan mode; mass spectra of the analytes in the mass charge (m/z) ratio range from 100 to 450 were recorded, and the scan range was narrowed down to a smaller range to improve the full scan sensitivity; in order to increase the selectivity and sensitivity, ions of the pesticides with higher mass ratios and abundances were preferentially selected as the precursor ions, and each precursor ion was examined to select the product ions in the daughter scan mode by applying collision energy voltages (0–35 eV) for fragmentation under collision–induced dissociation (CID) using argon gas as the collision gas; the collision energy (CE) was determined at the maximum product ion detection intensity for each pesticide, and the product ion with the highest signal intensity was selected for quantitative analysis , with the second transition used for confirmation.

The desolvation temperature and source temperature were set at 350 °C and 110 °C, respectively. The desolvation gas and cone gas were nitrogen (99.9995%) with flow rates of 500 L/h and 30 L/h, respectively. The collision gas was argon (99.999%) at a flow rate of 0.12 mL/min. The quantitative ion extraction chromatograms (EICs) of the 15 pesticides are shown in Figure S2.

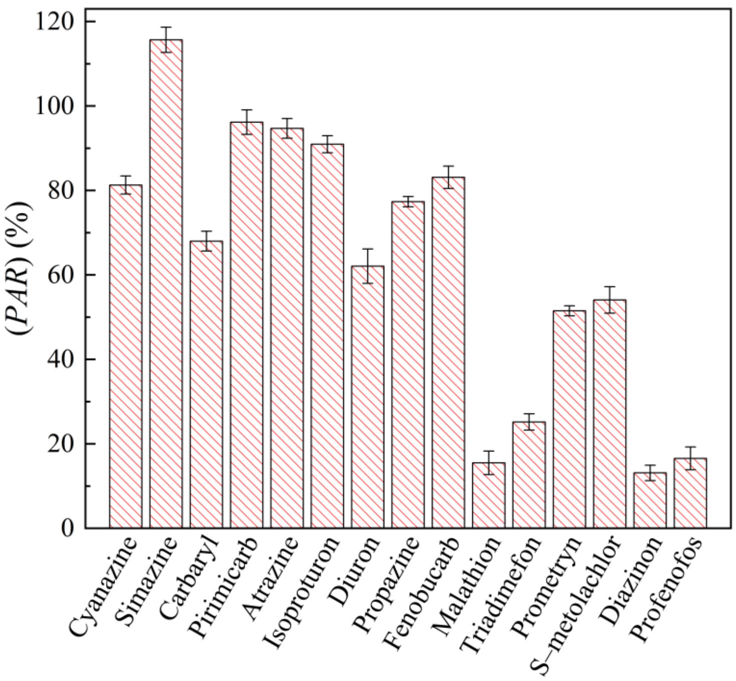

Figure S1 The ratio of the chromatographic peak area of the pesticide in UPW and 30% MeOH–UPW (2.5 μg/L).

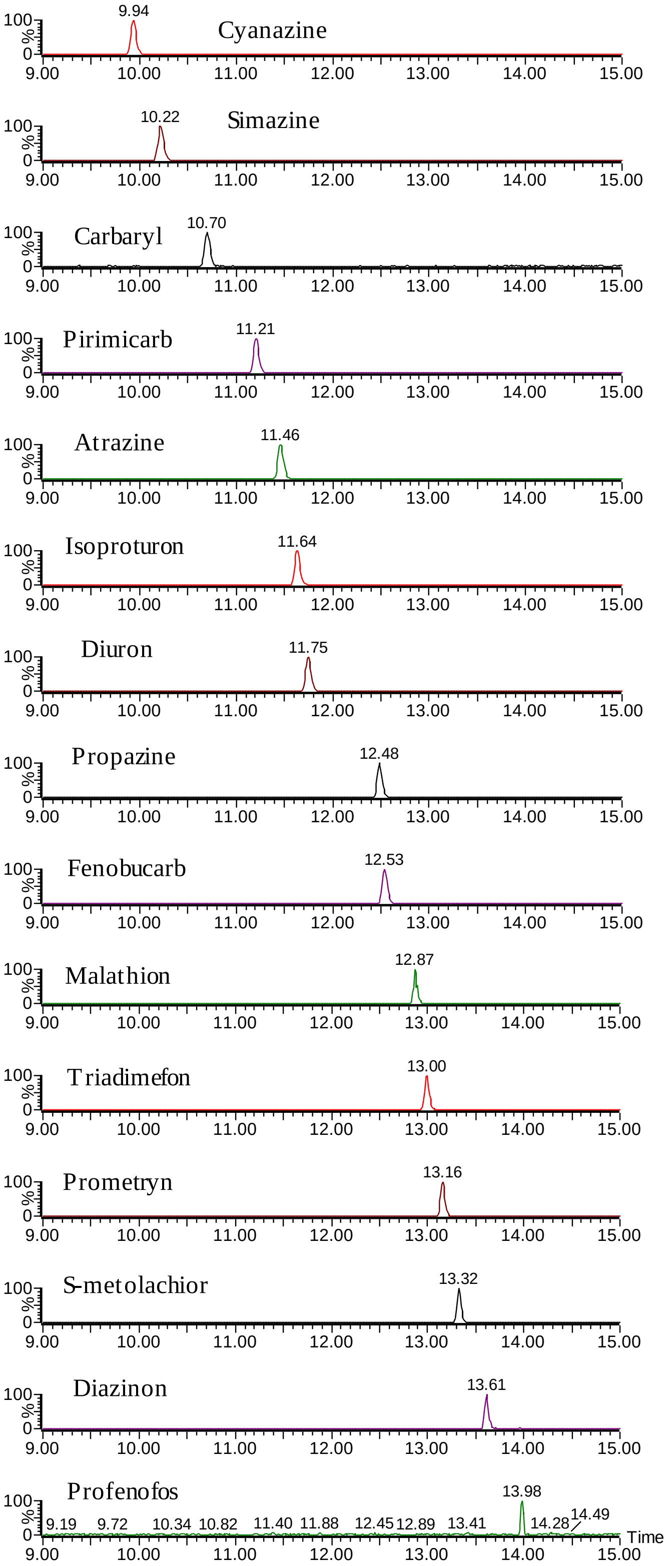

Figure S2 Quantitative ion extraction chromatograms of the 15 pesticides in the MRM mode (2.5 μg/L) (Note: The y–axis represents the relative response value of the peak height).

Table S1 The name, molecular formula, and structural formula of 15 pesticides.

**Analyte**	**Molecular formula**	**Structural formula**
Cyanazine	$C_9H_{13}ClN_6$	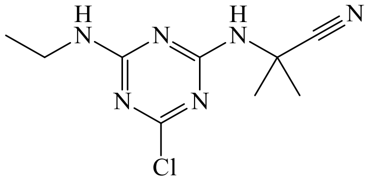
Simazine	$C_7H_{12}ClN_5$	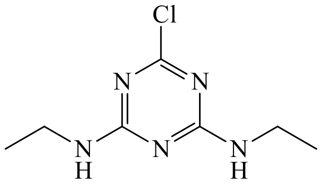
Carbaryl	$C_{12}H_{11}NO_2$	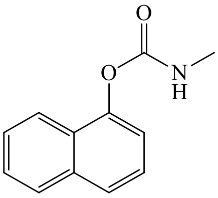
Pirimicarb	$C_{11}H_{18}N_4O_2$	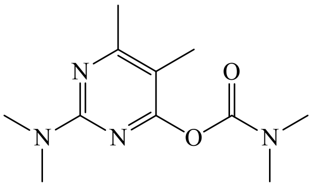
Atrazine	$C_8H_{14}ClN_5$	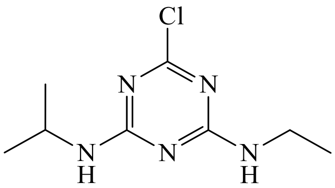
Isoproturon	$C_{12}H_{18}N_2O$	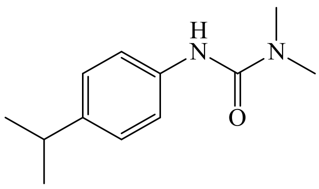
DCMU	$C_9H_{10}C_{l2}N_2O$	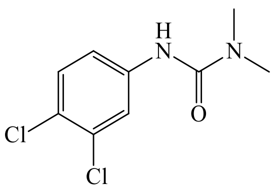

Analyte	Molecular formula	Structural formula
Propazine	$C_9H_{16}ClN_5$	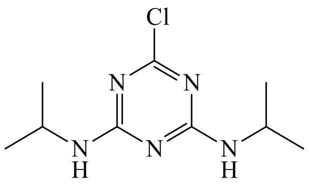
Fenobucarb	$C_{12}H_{17}NO_2$	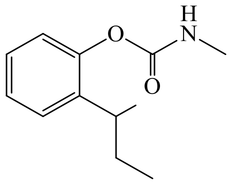
Malathion	$C_{10}H_{19}O_6PS_2$	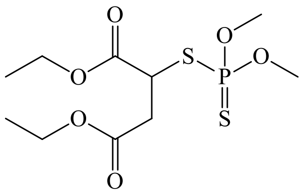
Triadimefon	$C_{14}H_{16}ClN_3O_2$	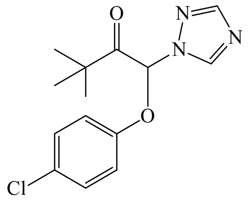
Prometryn	$C_{10}H_{19}N_5S$	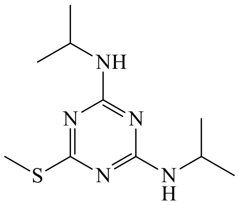
S–metolachlor	$C_{15}H_{22}ClNO_2$	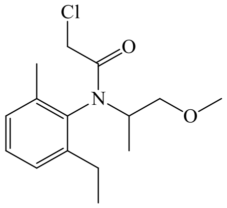
Diazinon	$C_{12}H_{21}N_2O_3PS$	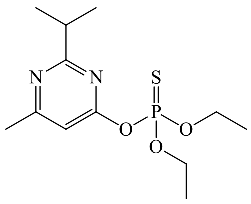
Profenofos	$CHBrClO_3PS$	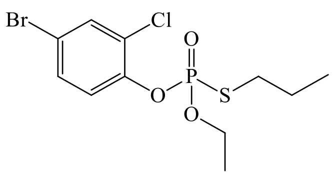

Table S2 The effect of 30% organic solvents on the AEs of the analytes in the glass injection vial (the detection signal intensity of the analyte in UPW was 100%).

Analyte	Relative intensity of detection signals (%)					
	UPW	30% MeOH–UPW	30% DMSO–UPW	30% IPA–UPW	30% ACN–UPW	30% ACE–UPW
Cyanazine	100.00±0.53	106.69±0.94	49.45±1.04	76.07±0.80	97.40±0.02	107.11±0.42
Simazine	100.00±1.24	99.49±0.18	43.80±1.53	66.14±0.25	91.24±0.20	94.13±0.02
Carbaryl	100.00±0.33	135.04±0.50	108.64±163	105.02±0.39	113.40±0.13	126.00±0.24
Pirimicarb	100.00±1.37	99.96±1.04	82.60±0.48	81.34±0.23	98.91±1.22	103.84±0.57
Atrazine	100.00±0.24	105.92±0.11	55.26±0.05	90.07±0.37	97.65±0.47	105.43±0.12
Isoproturon	100.00±0.32	113.14±0.55	121.75±0.12	102.23±0.10	108.51±0.32	112.95±1.69
DCMU	100.00±1.24	146.81±0.05	113.29±0.09	113.03±0.28	148.70±1.03	144.34±0.03
Propazine	100.00±0.70	112.34±0.36	64.01±0.22	80.85±0.05	104.54±0.54	114.23±1.05
Fenobucarb	100.00±0.95	115.11±1.28	98.95±0.74	90.32±0.49	110.12±0.09	114.42±0.12
Malathion	100.00±1.64	417.10±0.37	337.92±0.93	483.46±0.48	431.04±0.18	399.26±0.79
Triadimefon	100.00±1.18	223.57±0.75	136.86±0.34	164.65±1.20	205.59±0.07	222.71±0.07
Prometryn	100.00±0.90	173.44±0.03	142.07±0.77	145.35±1.73	166.22±0.09	168.13±0.06
S–metolachlor	100.00±2.23	190.35±0.29	172.33±0.20	159.79±0.11	186.23±1.69	197.31±0.79
Diazinon	100.00±1.12	488.00±0.16	425.00±1.02	420.79±0.18	511.80±0.77	598.88±0.13
Profenofos	100.00±2.81	938.89±0.83	405.56±1.93	988.89±1.58	972.22±0.80	127.22±0.61

Table S3 The effect of adding 30% organic solvents on the AEs of the 15 analytes in the plastic injection vial (the detection signal intensity of the analyte in UPW was 100%).

Analyte	Relative intensity of detection signals (%)					
	UPW	30% MeOH–UPW	30% DMSO–UPW	30% IPA–UPW	30% ACN–UPW	30% ACE–UPW
Cyanazine	100.00±1.13	102.03±1.41	40.54±0.50	63.42±1.82	94.89±0.47	102.36±0.09
Simazine	100.00±0.36	103.49±0.36	41.50±0.22	65.30±0.34	96.71±0.01	104.28±0.58
Carbaryl	100.00±1.08	139.71±0.08	82.63±0.20	71.43±0.2	124.94±0.13	143.12±0.40
Pirimicarb	100.00±0.70	102.27±0.95	82.67±0.01	83.32±0.21	107.99±1.18	104.75±1.05
Atrazine	100.00±1.07	102.62±0.07	50.74±0.09	82.65±1.27	104.79±0.26	108.63±0.48
Isoproturon	100.00±1.07	115.53±1.07	117.33±0.01	113.20±0.03	121.17±0.77	116.50±0.33
DCMU	100.00±0.87	153.99±0.27	117.89±0.05	126.99±0.25	151.96±0.05	151.38±0.28
Propazine	100.00±0.95	125.97±0.15	68.88±0.12	88.51±0.49	122.45±1.45	118.09±0.27
Fenobucarb	100.00±0.48	118.82±0.48	97.85±0.23	104.31±0.02	118.13±1.10	118.81±0.06
Malathion	100.00±2.18	719.10±0.36	692.47±0.83	748.97±1.35	697.60±0.20	984.25±0.29
Triadimefon	100.00±2.12	263.37±0.49	149.75±0.02	187.12±0.03	247.34±0.02	254.43±0.01
Prometryn	100.00±0.87	198.53±0.29	148.31±0.01	158.69±0.59	197.14±0.32	192.14±0.51
S–metolachlor	100.00±2.43	254.08±0.21	199.38±0.33	210.40±0.38	236.63±0.13	253.46±0.34
Diazinon	100.00±1.78	1356.11±0.56	809.05±0.23	1110.41±1.38	1307.24±1.32	1393.44±0.75
Profenofos	100.00±3.14	788.46±0.68	680.77±0.79	776.92±1.40	101.56±0.72	89.58±1.64

Table S4 Water-quality index of the real water samples.

Parameter	Surface water 1	Surface water 2	Ground water 1	Ground water 2
Turbidity (NTU)	5.43±0.11	10.22±0.18	2.43±0.11	7.72±0.003
pH	8.18±0.32	8.27±0.22	7.78±0.32	7.94±0.31
TOC (mg/L)	13.49±0.75	4.86±0.13	9.39±0.75	13.44±0.09
TC (mg/L)	53.71±2.13	32.87±1.11	45.61±1.06	55.91±1.84
IC (mg/L)	40.36±2.31	28.01±1.08	36.22±0.31	42.47±1.75
UV_{254} (cm^{-1})	0.19±0.085	0.086±0.001	0.04±0.085	0.02±0.003
NH_3-N (mg/L)	0.064±0.003	0.065±0.001	0.44±0.003	0.73±0.0001
Nitrate Nitrogen (mg/L)	6.65±0.24	2.31±0.12	18.65±0.24	25.98±0.08
Nitrite Nitrogen (mg/L)	0.005±0.0001	0.008±0.0001	0.56±0.0001	1.25±0.0003
TN (mg/L)	7.16±0.99	2.95±0.39	19.65±0.25	27.96±0.17
Phosphate (mg/L)	0.294±0.06	0.103±0.02	0.14±0.06	0.314±0.001
TP (mg/L)	0.34±0.009	1.034±0.11	0.24±0.009	0.79±0.001
Chloride (mg/L)	213.50±3.15	155.80±2.51	227.50±3.15	308.30±2.64
Sulfate (mg/L)	198.82±1.98	195.41±1.94	198.82±1.98	273.20±1.67
Total Hardness (Calculated by $CaCO_3$, mg/L)	232.51±3.21	300.57±4.26	432.51±3.21	560.14±4.89

Note: the water samples (Surface water 1, Surface water 2, Ground water 1 and Ground water 2) were taken from Yantai City (Shandong Province) and were taken from the Miaohou Reservoir, the Fenghuangshan Reservoir, Tashan Scenic Area, and Yantai Huili Town, respectively.

Table S5 Recoveries of the 15 pesticides added in the real water samples (30% MeOH–real water sample as solvent).

Analyte	Add pesticide concentration (μg/L)							
	0.25				2.5			
	Surface water 1	Surface water 2	Ground water 1	Ground water 2	Surface water 1	Surface water 2	Ground water 1	Ground water 2
Cyanazine	89.7±1.5	92.7±6.3	83.4±8.9	96.8±5.6	93.5±2.4	102.6±5.9	92.5±4.8	103.7±6.5
Simazine	85.5±2.4	98.5±3.7	105.1±3.5	106.5±3.8	90.3±8.9	94.6±2.6	98.6±2.4	97.5±2.8
Carbaryl	92.7±2.3	94.1±4.8	92.4±2.8	97.5±6.8	94.8±4.8	107.6±4.8	96.7±3.5	102.6±1.6
Pirimicarb	92.6±5.2	106.7±6.9	97.8±4.6	96.4±3.8	105.6±6.9	97.4±5.7	102.5±5.2	98.5±4.5
Atrazine	95.6±5.8	94.5±4.5	82.4±5.4	105.3±7.6	99.5±5.3	98.3±4.2	115.6±4.6	97.5±3.4
Isoproturon	105.7±8.6	102.7±2.2	94.8±4.4	96.4±5.2	92.3±1.5	98.4±3.7	103.6±5.4	103.8±5.7
DCMU	112.3±4.7	95.8±3.7	96.8±2.5	99.8±3.6	105.6±4.1	94.6±3.9	94.6±5.8	104.5±2.6
Propazine	94.8±3.9	115.6±5.8	97.5±6.4	96.7±5.9	102.6±3.7	98.5±5.7	101.5±2.3	103.4±3.5
Fenobucarb	103.6±2.5	97.5±4.9	98.6±4.8	115.5±7.6	97.2±2.8	106.8±3.2	96.7±1.6	94.8±4.8
Malathion	98.4±1.6	112.5±6.7	89.7±3.5	96.5±5.9	105.4±5.3	108.9±8.5	95.6±3.7	102.8±1.7
Triadimefon	95.6±4.4	87.8±3.4	114.6±6.2	121.8±6.4	96.1±5.9	90.6±4.3	103.4±6.5	106.7±5.6
Prometryn	92.5±7.8	102.5±1.7	98.6±7.8	88.7±8.5	94.6±6.7	98.7±3.4	96.8±4.8	102.5±5.8
S–metolachlor	89.7±8.6	99.7±3.2	116.5±5.7	82.6±10.8	103.8±2.9	102.9±2.6	96.4±2.8	92.4±3.8
Diazinon	103.6±1.2	98.4±2.5	93.5±3.9	93.5±6.2	97.5±7.2	103.5±2.3	94.8±3.4	97.5±2.6
Profenofos	80.6±9.8	108.6±8.5	91.8±9.2	112.7±9.5	84.5±9.8	92.7±8.4	103.8±9.8	98.6±8.1

Table S6 The name, retention time, and MRM conditions of the 15 pesticides.

Analyte	RT (min)	CV (V)	Quantitative transition			Confirmative transition		
			Precursor ion (*m*/*z*)	Product ion (*m*/*z*)	CE (eV)	Precursor ion (*m*/*z*)	Product ion (*m*/*z*)	CE (eV)
Cyanazine	9.94	33	240.89	213.86	15	240.89	85.76	25
Simazine	10.22	32	201.90	70.76	23	201.90	131.70	20
Carbaryl	10.70	43	144.83	126.81	17	144.83	116.80	17
Pirimicarb	11.21	30	238.96	71.72	25	238.96	181.90	15
Atrazine	11.46	35	215.91	173.80	17	215.91	103.70	25
Isoproturon	11.64	33	206.93	71.72	17	206.93	164.80	15
DCMU	11.75	30	232.82	71.79	17	232.82	45.70	15
Propazine	12.48	40	229.91	145.74	22	229.91	187.80	17
Fenobucarb	12.53	22	207.93	94.78	15	207.93	151.70	8
Malathion	12.87	27	330.86	126.75	11	330.86	98.70	18
Triadimefon	13.00	30	293.90	68.72	20	293.90	224.80	13
Prometryn	13.16	42	241.90	157.75	25	241.90	199.80	18
S–metolachlor	13.32	25	283.95	251.83	14	283.95	175.80	25
Diazinon	13.61	30	304.91	168.93	20	304.91	152.80	20
Profenofos	13.98	35	374.71	304.56	18	198.83	128.60	13
